# Predict Health Care Accessibility for Texas Medicaid Gap

**DOI:** 10.3390/healthcare9091214

**Published:** 2021-09-15

**Authors:** Jinting Zhang, Xiu Wu

**Affiliations:** 1 School of Resource and Environmental Science, Wuhan University, Wuhan 430079, China; whuzjt@whu.edu.cn; 2 Department of Geography, Texas State University, San Marcos, TX 78666, USA

**Keywords:** Medicaid gap, health access, principal components, logistical regression

## Abstract

Medicaid is a unique approach in ensuring the below poverty population obtains free insurance coverage under federal and state provisions in the United States. Twelve states without expanded Medicaid caused two million people who were under the poverty line into health insecurity. Principal Component-based logistical regression (PCA-LA) is used to consider health status (HS) as a dependent variable and fourteen social-economic indexes as independent variables. Four composite components incorporated health conditions (i.e., “no regular source of care” (NRC), “last check-up more than a year ago” (LCT)), demographic impacts (i.e., four categorized adults (AS)), education (ED), and marital status (MS). Compared to the unadjusted LA, direct adjusted LA, and PCA-unadjusted LA three methods, the PCA-LA approach exhibited objective and reasonable outcomes in presenting an odd ratio (OR). They included that health condition is positively significant to HS due to beyond one OR, and negatively significant to ED, AS, and MS. This paper provided quantitative evidence for the Medicaid gap in Texas to extend Medicaid, exposed healthcare geographical inequity, offered a sight for the Centers for Disease Control and Prevention (CDC) to improve the Medicaid program and make political justice for the Medicaid gap.

## 1. Introduction

**Figure 1:**
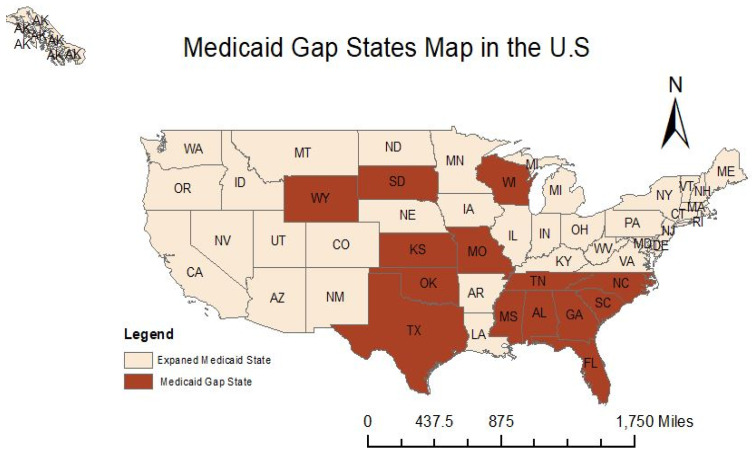
Medicaid gap map in the U.S.

Health care access is convoluted, emerging with different system constraints depending on the complex healthcare needs, improving access rests on system-targeted facilitators, interventions, and policies [1]. Despite the adoption of universal healthcare coverage (UHC) by member countries of the World Health Organization (WHO), the U.S. has the most expensive healthcare system in the world, comparatively [2]. Healthcare is a privilege for those who can afford it and is one of the most crucial issues in American society [3]. In fact, the American healthcare system is a hodgepodge of private and public insurance plans with cracks under the iron triangle of quality, cost, and access [4,5]. Increasing costs have forced many Americans more vulnerable to higher healthcare payments and potentially reduced access to care. Thus, healthcare is a top concern for those who are unable to access all the essential health services that they need. It is worth noting that providing scientifically explicit corroboration contributes to enlarged healthcare access. Medicaid is the largest common federal health care coverage program for low-income individuals of diverse ages in the United States and serves as a core institution that shapes public health crises, racial injustice, and electoral politics [6]. It was created by President Lyndon B. Johnson in 1965, playing a vital role in alleviating health inequities and reducing recidivism [7,8,9,10]. However, some regulations in Medicaid restrict eligibility, such as income, asset, and age requirement, dependent children limitation, and categorical eligibility (pregnant women, children, and disabled people). Those lead to the Medicaid gap is increased and should be considered. The Medicaid gap is defined as those who do not have private insurance and unqualified Medicaid requirements. They are exposed to high risks of health care, social security, as well as vulnerability. In 2012, the Supreme Court ruled to allow states to opt-out of the Medicaid expansion. In 2019, the Affordable Care Act (ACA) was advocated for the population who earned less than 138% of the federal poverty guidelines (FPG), improving healthcare access and quality while decreasing the expense of care [11,12]. By 2020, there have been 2.5 million Americans living in the Medicaid gap in 14 states without expanded Medicaid programs in Figure 1 [13]. In Texas, the Medicaid gap contains 20 million people, approximately 10% of the state’s population, being on one or more type of Medicaid [14]. Hence, better recognition of the ACA accessibility is beneficial to help those who are in the Medicaid gap address health inaccessibility, mitigate health conflictions, improve healthcare systems, and future healthcare policymaking.

**Figure 2:**
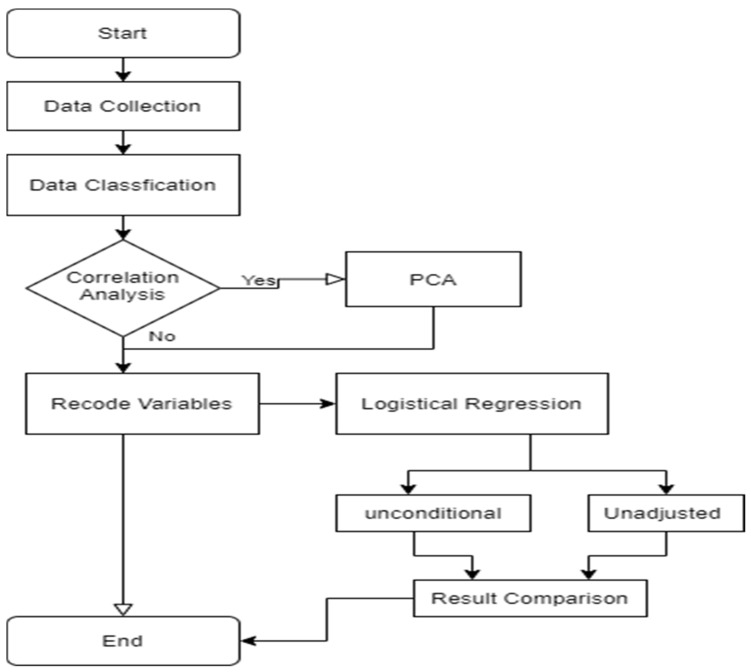
Study framework.

According to the Texas Medicaid yearly income limits standard, the Medicaid gap is defined as those whose income is less than USD 35,000 per family with one person (or USD 47,000 with two-person) without any insurance. Then, we conduct the inter-correlations among independent variables before performing PCA. If correlations exist, we extracted related factors of health status by PCA; otherwise, we directly recoded related independent variables to model the LR. Through the comparison of unconditional requirements and adjusted requirements, accordingly, the distinctions automatically revealed the pros and cons of the ACA policy in Texas. The study framework is in Figure 2.

## 2. Materials and Methods

### 2.1. Data

Data on 63,083 cases from 2013 to 2020, including 8 years of questionnaires in Table 1, were collected from The Behavioral Risk Factor Surveillance Survey (BRFSS), which is a national wide, random-digit-dial telephone survey. Investigation contents involved demographic information, health behaviors information, access ways to care, and health quo. This study obtained Medicaid gap data for all registered 18–64 years old people whose household income was less than 138% of the federal poverty guidelines without any insurance during 2013–2019 in Texas from the Center for Health Statistics in the Texas Department of State Health Services (DSHS). The Medicaid gap addresses of all cases were geocoded to their exact geographic locations (latitudes and longitudes) by the Texas DSHS.

In this study, health status is the subject of observation as a unique dependent variable, which has two values such as 0 (i.e., health “fair” or “poor”) and 1 (i.e., good health). Independent variables contain 14 variables, referring to health conditions, demographic data, education, and economic conditions four perspectives, as being seen in Table 2. Health conditions refer to “no regular source of care” (NRC), “last check-up more than a year ago” (LCT), “could not see a doctor due to cost” (NSD), “skipped medication due to cost” (SMC), “cardiovascular disease “(CVD), “diabetes” (DT), and “current smoker” (CS)—7 variables. Demographical data involve age structure, sex, race, and marital status. Economic conditions include employment (EM) and “living with dependent children” (LDC). Educations consist of “did not finish high school”, “high school graduate/GED”, and “college graduate”—three scenarios. Three medical types are distributed in diverse variables in Table 2.

### 2.2. Data Spatial Representation

**Figure 3:**
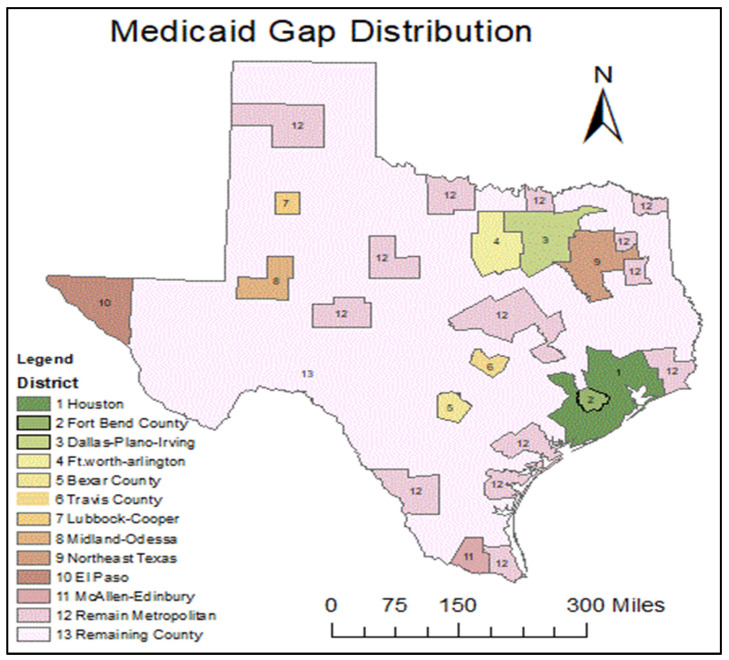
Medicaid Gap District distribution in TX.

In this study, study areas are categorized by above-poverty, Medicaid, and the Medicaid gap three insurance types in 13 areas in Texas in Figure 3.

### 2.3. PCA-LA Rationale

Logical regression is used for a broad range of research applications and is especially popular with medical research in which the dependent variable has a binary format outcome of either 0 or 1, true or false, yes or no, high or low. The general mathematical equation of logistical regression is written as follows, where *Y* is the dependent variable based on *X*_1_, *X*_2_ [15].

(1) $$Y=C+B_{1}X_{1}+B_{2}X_{2}+\ldots$$

In this research, we firstly standardized the matrix *X* with the independent variables into X*. Then, we extracted the *r* number of the principal components under the condition of *r* number of the sum of eigenvalue beyond 1. After that, the orthogonal matrix β with eigenvector was divided as β = (β1|β2), so the *r* number of values are presented as *Z*_1_ = X*β1, the following equation is calculated.

(2) $$Z_{j}=\sum_{i}^{r}l_{ij}X_{i}\;j=1,2,\;\ldots ,\;r$$

where *Z*_1_, *Z*_2_, …, *Z_r_* is viewed as diversely extracted factors. The relationship between factors equals 0 so that those factors are defined as independent variables. According to logistic regression this is a classification algorithm, so the assumption is that the coefficients of the factors equal to *a*_1_, *a*_2_, …, *a_r_*, the model is created as follows:

(3) $$P(y=1|z)=\frac{e^{a_{0}}+\sum_{i=1}^{r}\lambda_{i}X_{i}}{1+e^{a_{0}}+\sum_{i=1}^{r}\lambda_{i}X_{i}}$$

(4) $$\lambda_{i}=l_{i}{}^{r}a=l_{i1}a_{1}+l_{i2}a_{2}+\ldots +l_{ir}a_{r}\;i=1,\;2,\;\ldots ,\;p$$

We should conduct the Wald test after obtaining *λ_i_*. In light of SPSS statistical software (IBM, Armonk, NY, USA), the covariance matrix is calculated by the following equation.

(5) $$\left(\begin{array}{ccc} var\left(\alpha \right) & cov\left(\alpha_{1},\alpha_{2}\right) & \ldots \ldots cov\left(\alpha_{1},\alpha_{r}\right)\\ \ldots & \ldots & \ldots \\ cov\left(\alpha_{r1},\alpha_{1}\right) & cov\left(\alpha_{r},\alpha_{2}\right) & \ldots \ldots var\left(\alpha_{r}\right) \end{array}\right)$$

In light of the assumption of the likelihood ratio, $\alpha^{r}\;$= ($\alpha_{1},\alpha_{2},\ldots \ldots ,\alpha_{r})$ is met by a normal distribution. It leads to *λ* qualified for normal distribution that has characteristics of stationary in linear transformation. When the covariance of *λ* is captured, we will obtain the Wald test in the context of *λ* = 0 using the following equation.

(6) $$\mu_{i}=\frac{\lambda_{i}}{\sqrt{\mathrm{var}\left(\lambda_{i}\right)}}$$

If parts of independent variables are inter-correlated, we can just employ PCA to remove the association between a part of the variables based on the above steps [16]. The setting of the threshold value is a very important aspect of logistic regression, and 0.6 is defined as the threshold value in this study.

### 2.4. PCA-LA Analysis Procedure

The design of the study aims at eliminating multicollinearity between explanatory variables. The following procedure should be considered and ordered in sequence.

(1) Pearson correlation analysis in dependent variables. The bivariate Pearson correlation is used to estimate correlations among pairs of variables. The coefficients are revealed whether a statistically significant linear relationship exists between two continuous variables, as well as directions and strengths of a linear relationship. In the SPSS environment, the function of a bivariate is completed by choosing the correlate option of the analyzing menus [17].
(2) PCA to extract major components. Based on correlation analysis, we performed nine explanatory variables to extract component factors using PCA. The dataset was examined using Kaiser–Meyer–Olkin (KMO) and Bartlett’s Test of Sphericity. The KMO test compares the correlation statistics to identify if the variables include sufficient differences to extract unique factors. A KMO value of 0.56 for nine explanatory variables is more than the cutoff value of 0.7. The Bartlett’s Test of Sphericity (BTS) value of 0.0 was significant (*p* < 0.001), validating that correlation between variables does exist in the population. Communality is a common variance between 0 and 1, using the remaining variables as factors, which was used to determine if any variables should be excluded from the factor analysis. A 0.7 cutoff is used to determine the significance of explanatory variables [18]. Using an eigenvalue threshold greater than 1.0, four factors are identified that could explain a cumulative 64.7% of the variance after six iterations (Figure 3). A varimax rotation was used to assist in the interpretation of the PCA analysis. The rotated component matrix was examined for variables with a cutoff of 0.7.
(3) Perform logistical regression on major components. Geographic disparities in health care are well noted [19,20,21,22], but seldom research has shown healthcare spending varies by geographical location in the Medicaid gap via statistical analysis. Even some ethnic groups may be under-represented in the Medicaid expansion population because they are more likely to live in states that have not expanded Medicaid [23,24]. Current studies highlighted the policy adjustment from the qualitative analysis [25,26,27,28] except for Spencer et al. (2019). Despite the Medicaid Gap population in North Carolina for health access was conducted in Statistical inference, counter-comparative multi-variated indexes were not involved [29]. In addition, potential risks of chronic diseases were not estimated in the research. Most importantly, multicollinearity issues are not mentioned, which is a phenomenon that undermines the statistical significance of an independent variable, increases the standard deviation of variables, as well as the inverse direction of coefficients [30,31,32]. Logistical regression is generally popular in the application of epidemiology [33,34,35,36]; most research investigated models without eliminating multicollinearity among variables. PCA is one of the effective ways to reduce dimensionality and minimize multicollinearity. Currently published articles of logistical regression based on PCA are focused on genome-wide association studies [37] and disease research, such as gestational diabetes mellitus [38] and nephropathy [39]. Health care accessibility for the Texas Medicaid Gap took advantage of principal component analysis (PCA) to eliminate multicollinearity negative effects and to compare comprehensive social-economic impacts between unadjusted conditions and adjusted conditions. Therefore, PCA-based LA health access analysis of the medical gap is beneficial to provide scientific evidence regarding whether implementing ACA or not, to balance Medicaid policies in the U.S.
(4) T-tests and F-tests were used to validate the significance of the model. *T*-test compares the means of two independent groups in order to determine whether there is statistical evidence that the associated population means are significantly different. In SPSS, the “Independent-Samples *t*-test” function is selected in the “compare mean” default of analyzing menu [40]. In addition, the F-test can be used for determining whether the variances of two groups differ from each other. In the SPSS environment, “One-way analysis of variance (ANOVA Analysis)” is selected as “Homogeneity of variance test” in “compare means” of “options” [41].

## 3. Results

### 3.1. Sample Description

#### 3.1.1. Health Care Access

Medicaid gap has the biggest barrier in health condition among all insurance coverages, regarding the comparison between “bad” and “good” answers, including those in Medicaid gap were twice as likely to represent fair or poor health (72.5% versus 32.7% and 17.9%, *p* < 0.001); “no regular source of care” is 67.3%, higher than above-poverty (26%), lower than Medicaid (74.9%). In the performance of “last check-up more than a year ago”, the Medicaid gap is the highest one (20.1% above-poverty, 23.6% Medicaid, 54% Medicaid gap). In the investigation of “could not see a doctor due to cost “, the Medicaid gap is the highest one (23.4% above-poverty, 18.4% Medicaid, 51.9% Medicaid gap). In the investigation of “skipped medication due to cost”, the Medicaid gap is the number one (55.5% above-poverty, 48.9% Medicaid, 76.7% Medicaid gap). In “cardiovascular disease” comparison, the Medicaid gap took up the highest one (13.5% above-poverty, 10.2% Medicaid, 16.1% Medicaid gap). In the diabetes investigation, the Medicaid gap is the highest one (18% above-poverty, 31.7% Medicaid, 42.6% Medicaid gap). In the investigation of “current smoker”, the Medicaid gap is the number one (20.8% above-poverty, 11.9% Medicaid, 24.6% Medicaid gap).

#### 3.1.2. Demographic Status

The disparity of demographic in the Medicaid gap is more striking than others in three aspects. First, according to statistical investigations in Table 2, mostly, individuals in the Medicaid gap were younger (48.8 years) than either traditional Medicaid (59.9 years) or above-poverty Texan (54.0 years). The age distribution in the Medicaid gap is concentrated on those who are older than 25 and younger than 44. On the contrary, the age distribution in above-poverty and Medicaid are congregated on people who are beyond 55 years old. Second, the female population in the Medicaid gap (63.7%) is higher than either the above-poverty or Medicaid, even though the male weight of 36.3% is higher than 34.7% of the above-poverty group but lower than 45.3% of the Medicaid population. At last, the Hispanic population in Medicaid gap is the highest one (above-poverty 44.7%, Medicaid 31.3%, Medicaid gap 54%), compared to the non-Hispanic population.

#### 3.1.3. Economic, Educational, and Marital Status

The Medicaid gap has a higher vulnerability than either the above-poverty or Medicaid concerning economic, education and marital status. First, the Medicaid gap has a poor economic condition as regards two folds. Those who were unemployed in the Medicaid gap (49.5%) is higher than 45.8% Medicaid, lower than 69.7% of the above-poverty group. On the other hand, living with dependent children is reported the highest one among three groups (31.5% above-poverty, 18.5% Medicaid, 55.5% Medicaid gap). Second, “did not finish high school” in the Medicaid gap (37.3%) are higher than either the above-poverty (22.7%) or Medicaid (21.4%), meaning the Medicaid gap had lower educational attainment. Finally, 64.6% of single people in the Medicaid gap are lower than the above-poverty (67.9%) but higher than Medicaid (40%). The Medicaid gap has beyond a half portion of someone who experience loneliness, which results in inhibited either sociability problems [42] or mental health issues [43].

#### 3.1.4. Space–Time Sample Change

From a spatial distribution perspective, 63,083 samples are categorized into 3714 individuals in the Medicaid gap, 4615 individuals in Medicaid, and 54,754 individuals in above-poverty. The Metropolitan area accounted for 41,176 cases while the rural area accounted for 21,907 cases. Samples of this research relied on metropolitan observation. During 2013–2020, the Medicaid gap collected 4911 cases, whereas above-poverty had 6726 cases, as well as Medicaid who owned 51,446 cases. It indicates sample distribution is yearly unbalanced in three insurance types and Medicaid is more overweighted than other insurances in the procedure of sample collections. 

### 3.2. Correlation

Before checking correlations, all independent variables should be standardized to have a mean of 0 and a standard deviation of 1 (unit variance). In light of Table 3, 14 explanatory variables are significant to the dependent variable of HS due to a *p*-value less than 0.05. Nevertheless, if independent variables are not significant to each other, they also should be eliminated before conducting the PCA. In this study, sex, race, EM, CVD, and DT are not significant to connect other variables. They admittedly do not participate in modeling LR models. Meanwhile, the rest of the nine independent variables have correlated with each other due to coefficients not equal to zero. That is why we choose the PCA to create new uncorrelated variables that successively maximize variance. 

### 3.3. PCA Results

**Figure 4:**
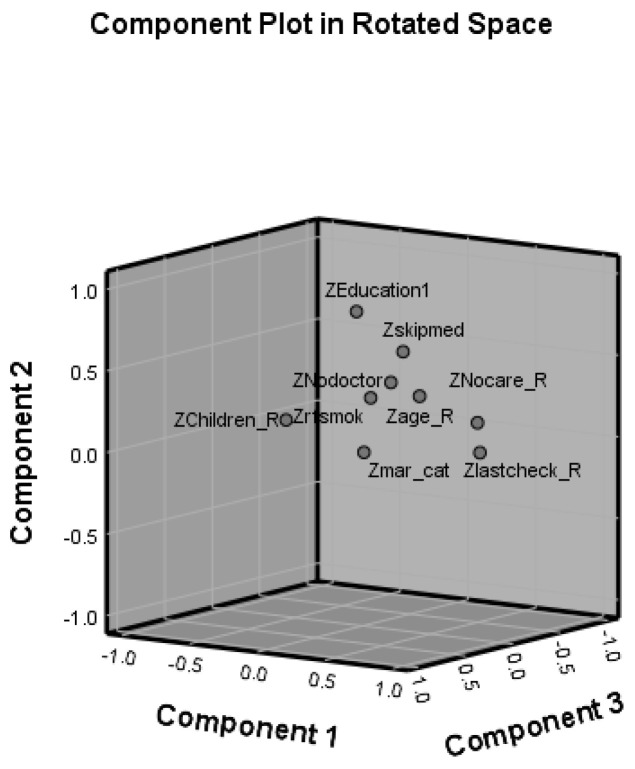
Component structure figure.

Table 4 gave us the direct relationship between factors and explanatory variables. The structure of components is shown in Figure 4. The first factor represents high loading on variables related to LCT and NRC, which have high similarities, indicating that HS is positively related to health conditions. Factor 2, called the education index, is positively related to the population who have reached various degrees. Factor 3 represents age structure, which is negatively related to HS, meaning older people have a worse health status. Factor 4 was the marital status index, positively affecting HS.

### 3.4. Logistical Regression Analysis

This research considered two scenarios in the context of three various insurance coverage conditions, consisting of logistical regression models based on PCA outcomes and direct logistical models. Each scenario also reflected diverse impacts on the independent variable in unadjusted or adjusted Medicaid policies. At the same time, it is readily to see a clear comparison between the PCA and the non-PCA model, and a discrepancy between unadjusted impacts and adjusted impacts via Table 5.

#### 3.4.1. Health Conditions

Through PCA, Factor 1 in Table 5 interpreted health condition changes regarding LCT and NRC. It is the most remarkable among factors. From the NRC perspective, despite the value in traditional Medicaid, it is not significant to health status owing to a *p*-value beyond 0.05, the *p*-values in beyond poverty and Medicaid gap are less than 0.01, indicating the value are statistically significant. In the beyond poverty group, the OR value equals 1.33 response to “no” as referent group in terms of “no regular source of care”, meaning those who had private insurances, but no regular check-up sources had a 133% higher chance of health risks when compared with people who had a regular source of care within private insurances conditions and a 95% confidence interval (CI). In the Medicaid gap, the OR value is 1.37 under CI 95%, implying those who did not have insurances and regular check-up sources had a 137% higher chance of health risks when compared with people who had a regular source of care in the Medicaid gap group. Most importantly, OR values under CI 95% are over 1 in beyond poverty and Medicaid gap situations, including two meanings. On one hand, an increase of “no regular source of care” results in a rise of the probability of health improvement. On the other hand, it is the outcomes of HS after adjustment tends to 1 (i.e., good health). At the same time, the OR value of 1.37 in the Medicaid gap and CI 95% is higher than the OR value of 1.33 in the beyond poverty group, portraying that the group of the Medicaid gap is more vulnerable than the group who have private insurances. Through comparisons of adjusted outcomes in having PCA and without PCA, the OR value of 3.29 of private insurance coverage in the direct LA model is higher than 1.33 in implementing PCA-LA, and 5.8 OR value of Medicaid gap in the direct LA model are higher than 1.37 in implementing PCA in response to referents, assuming that biased co-efficient estimates or very large standard errors for the logistic regression coefficients are hidden in the models [44]. From the LCT perspective, the difference of the LA model between having PCA and not having PCA is a direction change. When the OR value is less than 1 in the before PCA model, this means the LCT is negatively significant to HS and the values tend to be 1 (i.e., good health). Conversely, when the OR value is beyond 1 in the after PCA model, this means the LCT positively affects HS and the value tends to be 0 (i.e., “fair or poor” health).

#### 3.4.2. Demographic Impacts

Factor 2 after PCA in Table 5 is defined as the demographical adjustment of age structure. The referent is those whose age is less than 25. The predictors of AS under CI 95% are significant to HS in the above-poverty and Medicaid gap, due to a *p*-value less than 0.01. As in previous PCA analysis, AS is identified as a unique impact factor. The OR value under CI 95% is similar in the direct unconditional LA and the unadjusted PCA-LA, meaning recoding AS is not necessary. In the above-poverty group, the OR values of (0.08 in PCA-LA, 0.23 in the direct LA) those whose ages are older than 55 are the lowest OR values among the four age levels under CI 95%. It means that those who are beyond 55 years old are the most vulnerable group in the four various age groups. In the same vein, beyond 55-aged people are at the highest risk group of health care in the Medicaid gap. The OR values toward 0 of HS (i.e., “fair or poor” health), set forth that age structure increasing leads to a decline of the possibility of health risks after the adjusted insurance policy. When the OR value decayed, it displayed a linear attenuation regardless of insurance types.

#### 3.4.3. Education Impacts

Factor 3 in Table 5 points to education impacts. Those who are already college graduates are viewed as referents. All predictors of AS under CI 95% are significant to HS in three insurance types, except for PCA-LA in the Medicaid insurance group. According to PCA analysis, ED is extracted as a unique impact factor due to the beyond 0.7 threshold. The OR value under CI 95% is identical in the direct unconditional LA and the unadjusted PCA-LA, meaning recoding AS is not necessary. In three insurance types, the OR values in those who have not finished their high school degree or passed the GED are the lowest OR values, meaning they have the highest health risks results from an OR less than 1. The OR values less than 1 of HS (i.e., “fair or poor” health), delineated that education increasing triggers a decline of the possibility of health risks after the adjusted insurance policy. In addition, the Medicaid gap changes are more remarkable than the other two insurances after adjustment since the OR values are higher than the other two groups.

#### 3.4.4. Marital Status Impacts

Factor 4 in Table 5 refers to marital status impacts. Those who are married are regarded as referents. MS is a dichotomous independent variable. Interestingly, although the directly adjusted LA model is of no significance in the above-poverty and Medicaid gap, the PCA-LA is significant in the context of the three insurances. According to PCA analysis, MS is extracted as a unique impact factor due to the beyond 0.7 threshold. The OR value under CI 95% is identical in the direct unconditional LA and the unadjusted PCA-LA, meaning recoding MS is not necessary. After PCA analysis, the OR values in the three insurance types in the PCA-LA models are lower than the unadjusted OR, demonstrating it is essential to implement some other variables to control. Furthermore, those who are single had higher health risks compared to married people. The reason is that the OR value is less than 1. The OR values toward 0 of HS (i.e., “fair or poor” health), described marital status increasing stimulates a decline in the possibility of health risks after the adjusted insurance policy after the policy adjustment. In addition, the change after adjustment in the Medicaid gap is more remarkable than the other two insurances since the OR values are higher than the other two insurances. 

### 3.5. The Result of the T-Test and F-Test

In this research, Factors 1–4 are selected to conduct the T-test and F-test, respectively. Four components in the T-test have statistical significance owing to a *p*-value less than 0.05 and CI not across 0 value in Table 6. Four components in the F-test have statistical significance due to a *p*-value less than 0.05 in Table 7. We assumed that four main components are passed through the T-test and the F-test.

## 4. Discussion

This paper pinpointed the health access for the Medicaid gap, a multidimensional construct, going beyond its (mis)interpretation as a financial barrier in the existing political discourse on healthcare in the U.S. It addressed the relevance of understanding access for the Medicaid gap in the context of public health, expanding on the system levelers for change and potential approaches to drive change [45]. To begin with, the current problems in Texas are not just multiple chronic illnesses, but also health barriers. The Medicaid gap reports more multiple chronic conditions than the traditional Medicaid group in terms of two aspects. On one hand, the highest risk of multiple chronic conditions inferred that most of the Medicaid gap population is facing a more serious health crises without insurance coverage than the traditional Medicaid. On the other hand, they have been in the unhealthy status for a long time for lack of immediate therapies, which leads to complex medical needs. Notably, the Medicaid gap presented higher rates of access barriers than that of either the above-poverty group or the traditional Medicaid population, which directly leads to large, missed opportunities for preventive care of chronic diseases. There is a consensus of preventive services that could reduce health care expenses by early interventions to avoid disease worsening [46]. Second, economic hardship trip over the capability of the Medicaid gap. The Hispanic population makes up the highest percentage in Texas (50.4%), and females occupied 63.7% of the Medicaid gap, while the proportion of living with dependent children in the Medicaid gap is 55.5%, the highest among the three categories. Those in the Medical gap also account for the highest percentage (37.3%) to report not finishing high school. On other hand, the high unemployment rate in the Medicaid gap made them not afford self-sufficiency. Most importantly, those in the Medicaid gap population were reported having employment opportunities more than the traditional Medicaid population, while the percentage of those married in the Medicaid gap is higher than the traditional Medicaid gap. That illustrates they work hard for their family, ignoring their health benefit requirements. In addition, they pay attention to their earnings, rather than health benefits when looking for a job. After the expansion of Medicaid, the health access in the Medicaid gap is underlyingly broad in Texas. Especially, health warranty for the vulnerable population is dramatically improved.

By comparison of direct LA and PCA-LA, ACA adjustment in Texas could mitigate health pressure in the Medicaid gap and reduce health inequities [47]. First, health conditions directly have positive impacts on good health status in the Medicaid gap. Thanks to an OR value beyond 1, after the adjustment of the ACA, the Medicaid gap is bound to enter the Medicaid group so that some who do not have insurance could participate in the free insurance coverage system, saving more lives and reliving more suffering. Second, demographic impacts focus on beyond 55-aged elders who will have a decreased possibility of health risks after the adjustment. Third, education impacts are negatively related to HS, the adjustment will assist people who have not finished their high school degree in the Medicaid gap to have more opportunities to access the Medicaid system and reduce health risks. In addition, marital status has a negative influence on HS. Texas participating in the ACA program will benefit a single person in the Medicaid group, especially single women, to improve health care conditions and obtain more chances to access the Medicaid program.

In order to further acknowledge ACA impacts on the Texas healthcare system, we selected one state with the implementation of ACA, California, as a reference to compare the discrepancy of ACA impacts. Three main impacts should be noticed. First, California’s uninsured rate decreased 10%, and has remained at 7% until now. Since January 2021, the ACA in California has provided integrated health coverage to around one-third of Californians. Estimated 1.2–1.6 million people in California each year have been enjoyed healthcare benefits from the implementation of ACA every single year. (https://www.ppic.org/publication/health-care-reform-in-california/, accessed on 11 September 2021). Second, the implementation of ACA declined self-reported incapability to afford medical care and out-of-pocket spending, especially for low-income persons [48]. Lastly, the ACA implementation is associated with a reduction in financial risk and racial disparities [49]. 

Beyond the statistical significance of the Medicaid gap, we are further aware that the deep root of the decline of the Medicaid expansion is not just about financial limits, but also about the political contest. We called it the “Politics of Medicaid”, which means democratic states asserting Medicaid importance while republic states are holding Medicaid cuts [50]. When standing at marginal in a polity, the Medicaid gap has to stop to think about how policy and political vulnerability [51]. Indeed, the deep root relies on federalism. Medicaid is the epitome of how federalism produces inequity by constructing geographical disparity in access to vital healthcare resources [52]. As a boon, Medicaid is a program that saves thousands of lives each year and rendered tens of millions of Americans with free security of health insurance [53]. Geographical inequities between beneficiaries and the Medicaid gap are the product of policy choices made possible by federalism. Even though federalism is not good inherently, it is inevitable to see that federalism triggers mechanism injustice among economically and racially marginalized Americans and shapes the political effects of the Medicaid policy. 

Under the diversity of organizations in the healthcare system that exists in the world, Chinese healthcare system is more impressive than others. China has implemented a series of healthy campaigns to elevate the entire healthcare industry to international standards with global impacts, such as the Joint Commission International (JCI) and Healthcare Information and Management Systems Society (HIMSS). Those policies have been facilitated the comprehensive improvement of people’s health status and wellbeing [54] so that public healthcare coverage has been implemented to meet the requirement of universal access to basic care [55]. 

## 5. Conclusions

Although the Medicaid gap is marginal in the U.S, we sufficiently use quantitative methods to health access for Medicaid gap. Even though Medicaid is a political issue, this research is useful to local government, the Centers for Disease Control and Prevention (CDC) to estimate the Medicaid program implementation consequences by describing how their efforts in the long term can serve towards the improvement of health and socio-economic outcomes [56].

### 5.1. Limitation

This research has several limitations due to the healthcare statistic. First, based on the BRFSS dataset, this study has inevitable misclassifications when sorting out data. Medicaid samples are higher than the other two groups. A balanced number of samples will increase the accuracy of the analysis of the Medicaid gap. On the other hand, misclassifications are hidden in the standard of Medicaid. The FTP is defined as annual income between USD 20,000–21,404 in Texas. Due to the dynamic Texas FPG criteria, representative samples are divided into eight income categories. There are USD 1404 statistical errors in annual household income. In addition, one index covers many variables, such as multiple chronic conditions. Unmatched variable names may lead to missing data, affecting exact statistical results. Lastly, there are missing data in three variables in the statistic period. Variables missing may lead to the estimate value not being accurate. 

### 5.2. Implication

Based on conventional LA, the PCA-LA health access research for the Medicaid gap added PCA to eliminate multicollinearity disruption, emulated disparity of the Medicaid gap in Texas, visualized the domain concerns of healthcare, education, economic, and demographic impacts. Hence, it provides powerful evidence to improve the Medicaid program. The expansion of Medicaid, simultaneously, may result in both safer health behavior and outcomes for the Medicaid gap. Furthermore, the research quantified the contribution of government and the public sector, the roles of markets and the private sector, and the overlap of the public and private sector work [57]. Hence, assessing access to health services for the Medicaid gap could have a positive impact on the future health care of Texas. It also lays the groundwork for recognizing all the dimensions and complexities of healthcare access in Texas [58,59].

## Figures and Tables

**Table 1:** Medical type table by years.

Year	Above-Poverty	Medicaid	Medicaid Gap	Total
2013	638	6871	575	8084
2014	939	9912	874	11,725
2015	904	9481	764	11,149
2016	670	8070	509	9249
2017	732	8252	613	9597
2018	573	7370	472	8415
2019	2249	1483	1094	4826
2020	21	7	10	38
Total	6726	51,446	4911	63,083

Note: type 1—private insurance, type 2—Medicaid, type 3—Medicaid gap.

**Table 2:** The variables table for logistical regressions.

Predictors	Acronym	Value	AbovePoverty	Medicaid	Medicaid Gap	Interpretation
Health Condition						
No regular source of care	NRC	1	74.0%	25.1%	32.7%	No (Good)
No regular source of care		2	26.0%	74.9%	67.3%	Yes (Bad)
Last check-up more than a year ago	LCT	1	79.9%	76.4%	46.0%	No (Good)
Last check-up more than a year ago		2	20.1%	23.6%	54.0%	Yes (Bad)
Could not see doctor due to cost	NSD	1	76.6%	81.6%	48.1%	No (Good)
Could not see a doctor due to the cost		2	23.4%	18.4%	51.9%	Yes (Bad)
Skippedmedicationduetocost	SMC	1	44.5%	51.1%	23.3%	No (Good)
Skipped medication due to cost		2	55.5%	48.9%	76.7%	Yes (Bad)
Cardiovascular disease	CVD	1	13.5%	10.2%	16.1%	Yes (Bad)
Cardiovascular disease		2	86.5%	89.8%	83.9%	No (Good)
Diabetes	DT	1	18%	31.7%	42.6%	Yes (Bad)
Diabetes		2	82%	68.3%	47.4%	No (Good)
Current smoker	CS	1	79.2%	88.1%	75.4%	No (Good)
Current smoker		2	20.8%	11.9%	24.6%	Yes (Bad)
Demographic						
Age structure	AS	1	10.7%	3.9%	10.6%	Age (<=25)
Age structure		2	23.4%	12.6%	47.3%	25 < Age <= 44
Age structure		3	17.8%	9.4%	21.0%	44< Age <= 55
Age structure		4	48.1%	74.2%	21.2%	Age (>55)
Sex	SEX	1	34.7%	45.3%	36.3%	Male
Sex		2	65.3%	54.7%	63.7%	female
Race						
White	RACE	1	30.9%	50.0%	34.3%	White
Hispanic		2	44.7%	31.3%	54.0%	Hispanic
Black		3	20.8%	13.0%	8.8%	Black
Other		4	3.6%	5.7%	2.9%	Other
Economic condition						
Employment	EM	1	30.3%	54.2%	50.5%	Yes (Good)
Employment		2	69.7%	45.8%	49.5%	No (Bad)
Living with dependent children	LDC	1	68.5%	81.5%	44.5%	No (Good)
Living with dependent children		2	31.5%	18.5%	55.5%	Yes (Bad)
Education						
College graduate	ED	1	45.1%	44.2%	28.6%	
High school graduate/GED		2	32.1%	34.4%	34.1%	
Did not finish high school		3	22.7%	21.4%	37.3%	
Marital status	MS	1	32.1%	60.0%	35.4%	married
Marital status		2	67.9%	40.0%	64.6%	single
Health status		1	67.3%	82.1%	27.5%	good
Health status		0	32.7%	17.9%	72.5%	poor or fair

**Table 3:** Correlation table.

Predictors	Acronym	NRC	LCT	NSD	SMC	CS	AS	SEX	RACE	EM	LDC	ED	MS	CVD	DT
No regular source of care	NRC	1/0.00													
Last check up more than a year ago	LCT	−0.53/0.00													
Could not see doctor due to cost	NSD	0.25/0.00	0.24/0.00												
Skipped medication due to cost	SMC	0.10/0.00	0.01/0.04	0.27/0.00											
Current smoker	CS	−0.08/0.00	0.10/0.00	0.14/0.00	0.06/0.00										
Age Structure	AS	−0.17/0.00	−0.27/0.00	−0.16/0.00	−0.23/0.00	−0.03/0.00									
Sex	SEX	0.04/0.00	−0.07/0.00	0.11/0.00	0.02/0.00	−0.05/0.00	0.01/0.0.3								
Race	RACE	0.02/0.04	0.04/0.00	0.08/0.00	0.19/0.00	0.02/0.00	−0.15/0.00	0.01/0.1							
Employment	EM	0.09/0.00	−0.14/0.00	−0.02/0.00	0.14/0.00	−0.02/0.00	0.27/0.00	0.17/0.00	−0.03/0.00						
Living with dependent children	LDC	0.11/0.00	0.16/0.00	0.16/0.00	0.17/0.00	−0.00/0.03	−0.44/0.00	0.13/0.00	0.11/0.00	−0.17/0.00					
Education	ED	0.18/0.00	0.13/0.00	0.15/0.00	0.29/0.00	0.10/0.00	−0.04/0.00	0.04/0.00	0.13/0.00	0.06/0.00	0.16/0.00				
Marital status	MS	−0.07/0.00	0.47/0.00	0.10/0.00	0.17/0.00	0.12/0.00	−0.14/0.00	0.10/0.00	0.10/0.00	0.06/0.00	−0.09/0.00	0.06/0.00			
CVD	CVD	−0.07/0.00	0.07/0.00	−0.03/0.00	−0.03/0.00	−0.02/0.00	−0.09/0.00	0.05/0.00	−0.01/0.24	−0.15/0.00	0.05/0.00	−0.03/0.00	0.01/0.01		
Diabetes	DT	−0.21/0.00	0.02/0.00	−0.14/0.00	0.01/0.00	0.02/0.00	−0.06/0.00	−0.01/0.00	−0.13/0.00	−0.09/0.00	0.00/0.7	−0.23/0.00	0.03/0.00	0.16/0.00	
Health status	HS	−0.19/0.00	−0.04/0.00	0.22/0.00	0.20/0.00	0.05/0.00	−0.1/0.00	0.02/0.00	−0.1/0.00	0.12/0.00	0.04/0.00	−0.21/0.00	−0.11/0.00	−0.03/0.00	0.29/0.00

**Table 4:** Varimax with Kaiser Normalization Rotated principal component analysis.

Acronym	Extraction	Component			
		1	2	3	4
LCT	0.849	0.917	0.053	0.072	−0.010
NRC	0.878	0.903	0.236	0.078	0.032
ED	0.672	−0.030	0.816	0.023	−0.073
SMC	0.492	0.283	0.595	−0.021	0.236
NSD	0.274	0.257	0.417	0.070	0.171
AS	0.792	−0.168	0.159	−0.840	−0.185
LDC	0.765	−0.018	0.262	0.682	−0.289
MS	0.751	0.061	−0.031	0.074	0.861
CS	0.356	−0.029	0.265	−0.127	0.518

**Table 5:** Logistic regression analysis results—Medicaid gap.

			Unadjusted	Adjusted	PCA—Unadjusted	PCA—Adjusted
Variable/Factor	No. of Patients (%)	No. of Patients(N)	OR (95% CI) ^a^	aOR (95% CI) ^b^	aOR (95% CI) ^c^	aOR (95% CI) ^d^
No regular source of care						
Above-poverty						
NO—1	74.0%	4046	1.00 (referent)	1.00 (referent)	1.00 (referent)	1.00 (referent)
YES—2	26.0%	1419	1.9 [1.68, 2.19] *	3.29 [2.1, 5.12] *	1.7 [1.54, 1.9] *	1.33 [1.16, 1.53] *
Traditional Medicaid						
NO—1	25.1%	12,582	1.00 (referent)	1.00 (referent)	1.00 (referent)	1.00 (referent)
YES—2	74.9%	37,468	1.02 [0.97, 1.08]	1.56 [0.98, 2.48]	1.56 [0.97, 2.52]	1.18 [0.64, 2.17]
Medicaid Gap						
NO—1	32.7%	1561	1.00 (referent)	1.00 (referent)	1.00 (referent)	1.00 (referent)
YES—2	67.3%	3213	1.89 [1.66, 2.16] *	5.8 [4.01, 8.44] *	1.47 [1.27, 1.7] *	1.37 [1.18, 1.6] *
Last check-up more than a year ago						
Above-poverty						
NO—1	79.9%	5154	1.00 (referent)	1.00 (referent)	1.00 (referent)	1.00 (referent)
YES—2	20.1%	1294	0.36 [0.24, 0.54] *	0.35 [0.22, 0.55] *	1.7 [1.54, 1.9] *	1.33 [1.16, 1.53] *
Traditional Medicaid						
NO—1	76.4%	38,826	1.00 (referent)	1.00 (referent)	1.00 (referent)	1.00 (referent)
YES—2	23.6%	11,991	0.65 [0.61, 0.69] *	1.78 [0.67, 4.71]	1.56 [0.97, 2.52]	1.18 [0.64, 2.17]
Medicaid Gap						
NO—1	46%	2068	1.00 (referent)	1.00 (referent)	1.00 (referent)	1.00 (referent)
YES—2	54%	2427	1.07 [0.94, 0.1.22]	0.24 [0.17, 0.35] *	1.47 [1.27, 1.7] *	1.37 [1.18, 1.6] *
Could not see a doctor due to the cost						
Above-poverty						
NO—1	76.6%	5129	1.00 (referent)	1.00 (referent)	1.00 (referent)	1.00 (referent)
YES—2	23.4%	1567	0.45 [0.4, 0.5] *	0.60 [0.48, 0.75] *		
Traditional Medicaid						
NO—1	81.6%	1210	1.00 (referent)	1.00 (referent)	1.00 (referent)	1.00 (referent)
YES—2	18.4%	273	0.55 [0.4, 0.76] *	0.52 [0.35, 0.78] *		
Medicaid Gap						
NO—1	48.1%	2350	1.00 (referent)	1.00 (referent)	1.00 (referent)	1.00 (referent)
YES—2	51.9%	2539	0.37 [0.32, 0.42] *	0.38 [0.32, 0.44] *		
Age						
Above-poverty						
Age (<=25)—1	10.7%	718	1.00 (referent)	1.00 (referent)	1.00 (referent)	1.00 (referent)
Age (>25 and <=44)—2	23.4%	1574	0.26 [0.2, 0.35] *	0.44 [0.28, 0.67] *	0.26 [0.2, 0.35] *	0.26 [0.19, 0.35] *
Age (>44 and <=55)—3	17.8%	1197	0.11 [0.08, 0.14] *	0.30 [0.2, 0.47] *	0.11 [0.08, 0.14] *	0.11 [0.08, 0.15] *
Age (>55)—4	48.1%	3237	0.15 [0.11, 0.2] *	0.23 [0.15, 0.36] *	0.15 [0.11, 0.2] *	0.08 [0.06, 0.11] *
Traditional Medicaid						
Age (<=25)—1	3.9%	58	1.00 (referent)	1.00 (referent)	1.00 (referent)	1.00 (referent)
Age (>25 and <=44)—2	12.6%	187	0.0 [0.0, 0.0]	0.0 [0.0, 0.0]	0.0 [0.0, 0.0]	0.0 [0.0, 0.0]
Age (>25 and <=44)—3	9.4%	140	0.0 [0.0, 0.0]	0.0 [0.0, 0.0]	0.0 [0.0, 0.0]	0.0 [0.0, 0.0]
Age (>55)—4	74.2%	1105	0.0 [0.0, 0.0]	0.0 [0.0, 0.0]	0.0 [0.0, 0.0]	0.0 [0.0, 0.0]
Medicaid Gap						
Age (<=25)—1	10.6%	519	1.00 (referent)	1.00 (referent)	1.00 (referent)	1.00 (referent)
Age (>25 and <=44)—2	47.3%	2321	0.55 [0.42, 0.73] *	0.58 [0.42, 0.8] *	0.55 [0.42, 0.73] *	0.51 [0.38, 0.69] *
Age (>25 and <=44)—3	21.0%	1032	0.28 [0.21, 0.37] *	0.41 [0.29, 0.57] *	0.28 [0.21, 0.37] *	0.28 [0.2, 0.38] *
Age (>55)—4	21.2%	1039	0.21 [0.16, 0.28] *	0.32 [0.22, 0.45] *	0.21 [0.16, 0.28] *	0.18 [0.14, 0.25] *
Education						
Above-poverty						
Education (College Graduate)	45.1%	3033	1.00 (referent)	1.00 (referent)	1.00 (referent)	1.00 (referent)
Education (High School Graduate/GED)	32.1%	2157	0.61 [0.54, 0.69] *	0.81 [0.63, 1.02]	0.61 [0.54, 0.69] *	0.8 [0.68, 0.93] **
Education (Did not finish High School)	22.7%	1528	0.42 [0.37, 0.48] *	0.71 [0.55, 0.92] *	0.42 [0.37, 0.48] *	0.63 [0.53, 0.75] *
Traditional Medicaid						
Education (College Graduate)	44.2%	658	1.00 (referent)	1.00 (referent)	1.00 (referent)	1.00 (referent)
Education (High School Graduate/GED)	34.4%	513	0.7 [0.51, 0.96] **	0.62 [0.42, 0.92] *	0.7 [0.51, 0.96] **	0.86 [0.48, 1.54]
Education (Did not finish High School)	21.4%	319	0.61 [0.43, 0.87] *	0.6 [0.38, 0.95] *	0.61 [0.43, 0.87] *	0.62 [0.33, 1.17]
Medicaid Gap						
Education (College Graduate)	28.6%	1400	1.00 (referent)	1.00 (referent)	1.00 (referent)	1.00 (referent)
Education (High School Graduate/GED)	34.1%	1673	0.87 [0.74, 1.03]	0.84 [0.68, 1.02]	0.87 [0.74, 1.03]	0.88 [0.73, 1.06] *
Education (Did not finish High School)	37.3%	1830	0.62 [0.53, 0.73] *	0.63 [0.52, 0.78] *	0.62 [0.53, 0.73] *	0.72 [0.6, 0.86] *
Marital Status						
Above-poverty						
Married—1	32.1%	2147	1.00 (referent)	1.00 (referent)	1.00 (referent)	1.00 (referent)
Single—2	67.9%	4550	0.69 [0.61, 0.77] *	0.88 [0.71, 1.1]	0.69 [0.61, 0.77] *	0.63 [0.55, 0.72] *
Traditional Medicaid						
Married—1	60.0%	30,769	1.00 (referent)	1.00 (referent)	1.00 (referent)	1.00 (referent)
Single—2	40.0%	20,504	1.54 [1.47, 1.61] *	0.61 [0.39, 1.0] **	1.54 [1.47, 1.61] *	0.52 [0.31, 0.88] **
Medicaid Gap						
Married—1	35.4%	1731	1.00 (referent)	1.00 (referent)	1.00 (referent)	1.00 (referent)
Single—2	64.6%	3155	1.0 [0.89, 1.15]	0.93 [0.78, 1.09]	1.0 [0.89, 1.15] *	0.85 [0.73, 0.98] **

* *p* < 0.001; ** *p* < 0.05. Note: ^a^—not adjusted for any other variable; ^b^—adjusted for all other variables simultaneously; ^c^—not adjusted factors in PCA; ^d^—adjusted for all other factors simultaneously.

**Table 6:** T-test results.

Components	Equal Variances	t	df	Sig.	Lower CI	Lower CI
Health conditions	assumed	−4.029	4103	0.000	−0.224	−0.077
	not assumed	−3.906	2099	0.000	−0.227	−0.075
Demographic	assumed	8.987	4103	0.000	0.212	0.33
	not assumed	9.068	2287	0.000	0.212	0.33
Education	assumed	−13.368	4103	0.000	−0.504	−0.375
	not assumed	−13.151	2164	0.000	−0.505	−0.374
Marital Status	assumed	2.143	4103	0.032	0.006	0.14
	not assumed	2.113	2176	0.035	0.005	0.142

**Table 7:** F-test Results.

Components	Linear Term	df	Mean Square	F	Sig.
	Combined	716	0.352	1.998	0.000
Health conditions	Weighted	1	3.350	18.990	0.000
	Deviation	715	0.348	1.974	0.000
Demographic	Weighted	1	16.408	93.014	0.000
	Deviation	715	0.330	1.871	0.000
Education	Weighted	1	35.476	201.105	0.000
	Deviation	715	0.303	1.720	0.000
Marital Status	Weighted	1	0.950	5.386	0.000
	Deviation	715	0.352	1.993	0.000

## Data Availability

All data, models, and code generated or used during the study appear in the submitted article.
